# Small extracellular vesicles: a novel drug delivery system for neurodegenerative disorders

**DOI:** 10.3389/fnagi.2023.1184435

**Published:** 2023-06-19

**Authors:** Renjie Pan, Dongdong Chen, Lanlan Hou, Rong Hu, Zhigang Jiao

**Affiliations:** ^1^First Clinical Medical College, Gannan Medical University, Ganzhou, Jiangxi, China; ^2^Laboratory Medicine, First Affiliated Hospital of Gannan Medical University, Ganzhou, Jiangxi, China; ^3^Precision Medicine Center, First Affiliated Hospital of Gannan Medical University, Ganzhou, Jiangxi, China; ^4^Key Laboratory of Prevention and Treatment of Cardiovascular and Cerebrovascular Diseases, Ministry of Education, Gannan Medical University, Ganzhou, Jiangxi, China

**Keywords:** small extracellular vesicles, drug delivery system, neurodegenerative diseases, brain targeting, blood–brain barrier, treatment

## Abstract

Neurodegenerative diseases (NDs) have a slow onset and are usually detected late during disease. NDs are often difficult to cure due to the presence of the blood–brain barrier (BBB), which makes it difficult to find effective treatments and drugs, causing great stress and financial burden to families and society. Currently, small extracellular vesicles (sEVs) are the most promising drug delivery systems (DDSs) for targeted delivery of molecules to specific sites in the brain as a therapeutic vehicle due to their low toxicity, low immunogenicity, high stability, high delivery efficiency, high biocompatibility and trans-BBB functionality. Here, we review the therapeutic application of sEVs in several NDs, including Alzheimer’s disease, Parkinson’s disease, and Huntington’s disease, discuss the current barriers associated with sEVs and brain-targeted DDS, and suggest future research directions.

## 1. Introduction

Neurodegenerative diseases (NDs) are a group of chronic progressive diseases characterized by loss of neurons or myelin sheaths, mainly including Alzheimer’s disease (AD), Parkinson’s disease (PD), and Huntington’s disease (HD). The prevalence of NDs is closely related to age and has increased significantly with the accelerated aging of the population. The pathological and molecular mechanisms underlying these NDs are still unknown and need to be investigated. Furthermore, the blood–brain barrier (BBB) in the central nervous system (CNS), a highly selective and semi-permeable barrier, has created great challenges for treating and diagnosing NDs (Chen J. J. et al., 2019). Currently, an increasing number of diagnostic and therapeutic modalities are being used to explore this group of diseases. For example, Yu et al. (2020) found that cell-penetrating peptide (TAT) labeling of vasoactive intestinal peptide (VIP) could positively allosterically modulate the neuropeptide receptor PACAP type 1 receptor (PAC1) and enhance VIP neuroprotection in a mouse model of 1-methyl-4-phenyl-1,2,3,6-tetrahydropyridine (MPTP) in PD, which may be useful for developing therapeutic agents for ND. Moreover, Ren et al. (2013) found that implantation of mesenchymal stem cells (MSCs) with glial cell-derived neurotrophic factor (GDNF)-expressing MSCs into the striatum and substantia nigra improved motor function in PD monkeys, whereas transplantation of MSCs alone did not, suggesting that MSCs may be delivery vehicles for delivering GDNF to enhance nigrostriatal function. However, almost all large molecule drugs and >98% of small molecule drugs have failed in hybridizing the BBB, with candidates exhibiting poor biopharmaceutical and pharmacokinetic properties (Zhang et al., 2016). Drug delivery systems (DDSs) are formulations or technologies that enhance the safety and efficacy of drugs by controlling the rate, timing, and location of drug release *in vivo*. In clinical practice, DDS can be used to achieve precise controlled release of drugs, improve drug targeting and water solubility, regulate drug metabolism time, and promote drug bio-barrier penetration and absorption (Vader et al., 2016). Therefore, DDS are considered an effective treatment approach for these NDs, and suitable DDS that do not interfere with healthy organs and tissues are urgently needed to distribute drug molecules.

In the past decades, many researchers have explored DDS for the treatment of NDs, which include liposomes (Qu et al., 2018a) polymeric nanoparticles (PNPs) (Zhao Y. et al., 2020), solid lipid nanoparticles (SLNs) (Dal Magro et al., 2017), nanogels (Neamtu et al., 2017), and nanoemulsions (Zhao et al., 2018). Indeed, Qu et al. (2018a) designed a brain-targeted DDS using a 29-amino acid peptide (RVG29) from rabies virus glycoprotein (RVG) as a targeting ligand and found that RVG29-lip showed significantly higher uptake efficiency in mouse brain endothelial and dopaminergic cells and penetrated the BBB *in vitro* with high efficiency, effectively treating PD. Moreover, Zhao Y. et al. (2020) used poly(ethylene glycol)-co-poly(ε-caprolactone) (PEG-PCL) nanoparticles to encapsulate ginkgolide B (GB) prior to slow release into the bloodstream to treat PD. Although some of these DDS have been used clinically, there are still some problems, such as high toxicity (Niu et al., 2019), high immunogenicity (Chu et al., 2020), poor stability (Frank, 1993; Chen Z. J. et al., 2020), and low delivery efficiency (Prikhozhdenko et al., 2021). Small extracellular vesicles (sEVs) are considered the key to solving these problems and represent the most promising DDS. The reader is referred to the many previous reviews about the advantages and disadvantages of these DDS (Table 1).

**TABLE 1:** Comparison of the advantages and disadvantages of drug delivery strategies.

Strategy	Advantages	Disadvantages	References
Liposomes	Easy to load, high biodegradability, low toxicity	Short drug duration, short storage time	Frank (1993), Wong et al. (2012)
Polymeric nanoparticles	Biocompatible, low toxicity, high tolerability, high stability	Particle aggregation, nanotoxicity	Chan et al. (2010), Niu et al. (2019)
Solid lipid nanoparticles	High biodegradability, biocompatible	Easily cleared by the reticuloendothelial system	Dal Magro et al. (2017), Li X. et al. (2017)
Nanogels	Biocompatible, high stability	Possibility of toxic residues	Dal Magro et al. (2017), Li X. et al. (2017)
Nanoemulsions	High stability, high bioavailability	Thermodynamic instability	Dal Magro et al. (2017), Neamtu et al. (2017), Yukuyama et al. (2017)
Small extracellular vesicles	Biocompatibility, high stability, low immunogenicity, high specificity	Short cycle time, lack of standardized separation and purification methods, low drug loading efficiency, insufficient clinical-grade production	Meng et al. (2020), Parada et al. (2021)

**FIGURE 1:**
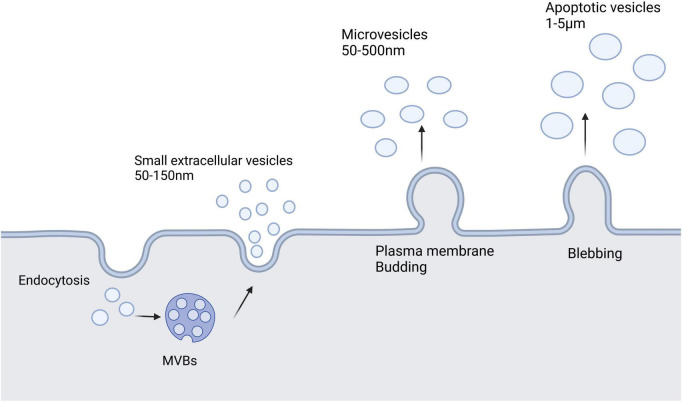
Extracellular vesicles secreted by cells mainly include three types: small extracellular vesicles, microvesicles, and apoptotic vesicles. Small extracellular vesicles are formed by the endosomal pathway of cells. Microvesicles are larger than small extracellular vesicles and result from the formation of outward buds in the plasma membrane. Apoptotic vesicles are bubble-like bodies produced during apoptosis.

Extracellular vesicles (EVs) are a heterogeneous group of cell-derived membrane structures that are found in biological fluids and are involved in various physiological and pathological processes. EVs are now considered as an additional mechanism for intercellular communication, which allow cells to exchange proteins, lipids, and genetic material (van Niel et al., 2018). EVs can be classified in many ways, but depending on their size, biological properties, biogenesis, and release process, they can be broadly classified into three categories: sEVs, microvesicles, and apoptotic vesicles. sEVs are generally 50–150 nm in diameter. Membrane invaginations of vesicles can form multivesicular bodies (MVBs), some of which can fuse with the plasma membrane and release internal vesicles to form sEVs by exocytosis, whereas other MVBs are degraded by lysosomes. Microvesicles have a diameter of 50–500 nm, even up to 1 μm, and they are derived directly from the cell membrane through budding, whereas apoptotic vesicles are usually produced by apoptotic cells and are approximately 1–5 μm in diameter (van Niel et al., 2018; Han et al., 2022). Additionally, EVs are heterogeneous in terms of content, with sEVs and microvesicles known to contain mRNA, miRNA, non-coding RNAs, and cytoplasmic and membrane proteins, whereas apoptotic vesicles contain nuclear fractions and cell organelles (Abdik et al., 2019). Figure 1 shows a schematic diagram of the three vesicle release methods.

Due to their natural origin, EVs are inherently highly biocompatible, with enhanced stability and limited immunogenicity, which offers potential advantages over traditional drug delivery vehicles such as liposomes and nanoparticles (Meng et al., 2020). Therefore, EVs are also considered as DDS (Elsharkasy et al., 2020). However, the DDS in this paper refers mainly to sEVs rather than microvesicles and apoptotic vesicles, the physicochemical properties of which make them unsuitable as DDS (De Jong and Borm, 2008). sEVs were selected as DDS for three reasons: first, sEVs carry and protect a wide range of biomolecules and can deliver them to recipient cells; second, they can cross the BBB and are distributed with some stability in the circulation, thus reaching different organs; and third, they can be designed to optimize delivery to certain tissues and have a high level of biocompatibility (Elsharkasy et al., 2020). Therefore, we focused on the application of sEVs as DDS, the biochemical properties of sEVs, and the applications of modified sEVs. sEVs can transfer proteins and genetic material in both directions between neurons and glial cells. Such cell-cell interactions contribute to the pathological progression and spread of NDs (Pinnell et al., 2021). Hence, we also discuss recent research advances in the use of sEVs for therapy of NDs, the limitations of sEVs as a DDS for the treatment of NDs, and the main directions for future research.

## 2. Biochemical properties of sEVs

### 2.1. Biogenesis of sEVs

**FIGURE 2:**
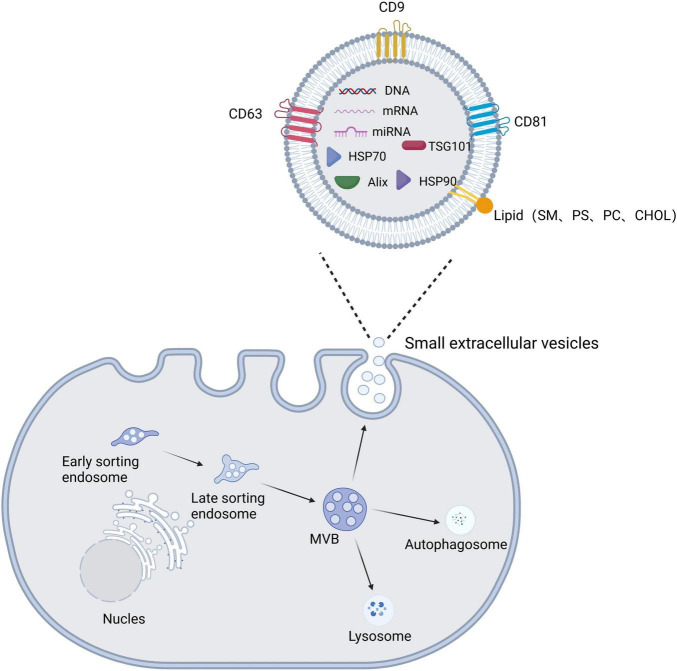
Schematic diagram of the biogenesis, secretion, and structural composition of small extracellular vesicles. The plasma membrane invaginates to form early sorting endosomes, which undergo continuous maturation to form late sorting endosomes and eventually increase multivesicular bodies (MVBs). MVBs have two main fates: they are fused by autophagosomes or lysosomes, or they fuse with the plasma membrane to release the contained intraluminal vesicles (ILVs) as small extracellular vesicles. Small extracellular vesicle structures contain proteins, lipids, and nucleic acids.

The production of sEVs involves dual invasion of the endosomal pathway and the plasma membrane (Kalluri and LeBleu, 2020). First, membrane proteins and extracellular soluble molecules invade the plasma membrane to form early sorting endosomes (ESEs), which then continue to mature to form late sorting endosomes (LSEs). Next, the LSE membrane invaginates to produce intracellular MVBs containing intraluminal vesicles (ILVs) (Li M. et al., 2022). MVBs have two main fates: they are fused by autophagosomes or lysosomes before being degraded and then recycled by the cell, or they are translocated to the plasma membrane, where they fuse with the plasma membrane and release ILVs via cytosolic vesicles, termed sEVs (Figure 2; Kalluri and LeBleu, 2020; Teng and Fussenegger, 2020; Gurung et al., 2021).

### 2.2. Composition and function of sEVs

Small extracellular vesicles are composed mainly of lipids, nucleic acids, and proteins. sEVs have a lipid bilayer composed mainly of plasma membrane lipids, such as sphingomyelin (SM), desaturated phosphatidylethanolamine, phosphatidylserine (PS), desaturated phosphatidylcholine (PC), cholesterol (CHOL), GM3, and ganglioside (Subra et al., 2007; Lu et al., 2022). The lipid bilayer serves a protective and drug-carrying function, with sphingomyelin and cholesterol adding stiffness to maintain the structural stability of sEVs (Kooijmans et al., 2012). The nucleic acid component of sEVs consists mainly of DNA, mRNA, miRNA, and other non-coding RNAs that mediate intercellular communication and regulate cellular function (Men et al., 2019). sEV proteins include constitutive proteins such as tetraspanin proteins (e.g., CD9 and CD63), which can be used to identify sEVs, and cell type-specific proteins, such as histocompatibility complex (MHC) class II expressed on MHC class II cells (Thery et al., 2002). High expression of transmembrane proteins (especially CD9, CD63, and CD81) and miRNAs (e.g., miR-124, miR-132, and miR-212) confirm the targeting function of sEVs (Song et al., 2019). For example, Zhdanova et al. (2021) found labeled sEVs in the hippocampus and neocortex after intranasal administration of sEVs expressing the typical markers CD9, CD63, and CD81 in a mouse model of AD. Lee et al. (2017) injected sEVs showing high expression of miR-219 into the striatum of R6/2 transgenic HD mice, which reduced expression of the target gene RE1-silencing transcription factor, demonstrating the targeted delivery of miR-219 based on sEVs.

### 2.3. Separation techniques for sEVs

The isolation and purification of sEVs as a carrier for DDS is critical. Common separation methods include ultracentrifugation, density gradient centrifugation, ultrafiltration, volume exclusion chromatography (SEC), immunoaffinity capture, and polymer precipitation (Li C. et al., 2022); the advantages and disadvantages of these methods are listed in Table 2. In addition, numerous researchers have explored the combination of several different separation methods. For example, Shu et al. (2020) combined ultrafiltration and SEC in cell culture media and found that their combination produced up to 58-fold more sEVs than ultracentrifugation alone. Koh et al. (2018) also found that sEVs were more efficiently enriched by combining the use of ultracentrifugation and SEC compared to the two methods alone. Although more than two separation methods increase the yield of sEVs to some extent, they can greatly increase the experimental time, which warrants the development of more effective separation methods. In an attempt to do so, Visan et al. (2022) found that tangential flow filtration reduced the time consumption for separating sEVs before SEC and greatly improved the recovery of sEVs compared to ultracentrifugation. Furthermore, Zhao et al. (2016) developed a microfluidic device (sEVsDEP chip)-based method for the separation and detection of sEVs, enabling microsphere-mediated dielectrophoretic separation and immunoaffinity detection. This approach allows the integration of different functions into the chip, such as downstream analysis to detect the main components of sEVs (Zhao et al., 2016). Additionally, microfluidic chips have the advantage of continuous separation and automation. Although many methods have been established for isolating and purifying sEVs, the main problem faced at this stage is the lack of standardized techniques for separating sEVs that preserve their structural and functional integrity, and the need to select different separation methods for different purposes and applications or develop other approaches. With the development of scientific technology, we believe that more applicable devices will appear in the future and/or researchers will format a new guideline when sEVs have been studied in depth.

**TABLE 2:** Comparison of the advantages and disadvantages of sEV separation methods.

Methods	Principle	Advantages	Disadvantages	References
Ultracentrifugation	Separation based on size and density differences between sEVs and extracellular contents	Simple method and simple sample preparation	Time-consuming, low-throughput, low-purity, compromised sEVs	Visan et al. (2022), Xu et al. (2022)
Density gradient centrifugation	According to the different sedimentation coefficients of different particles, they are separated under the action of centrifugal force	High purity and integrity	Low volume, time-consuming, high cost, not suitable for mass production	Chen B. Y. et al. (2019), Veerman et al. (2021)
Ultrafiltration	Membrane separation based on vesicle size	Time-saving and low cost	Stress of squeezing may disrupt the structural integrity of sEVs	Yang et al. (2020), Shu et al. (2021)
Size-exclusion chromatography	Isolation of sEVs from other extracellular vesicles based on size	Mild method in which the biological properties and structure of sEVs are well preserved	Time-consuming, high cost, low yield	Yang et al. (2020), Shu et al. (2021), Veerman et al. (2021), Visan et al. (2022)
Immunoaffinity capture	Isolation of sEVs based on antigen-antibody reaction	High purity and high specificity	High cost, antibodies subject to non-specific binding, not suitable for large-scale production	Mondal and Whiteside (2021), Veerman et al. (2021), Xu et al. (2022)
Polymer precipitation	Isolation of sEVs based on reduced solubility	Simple operation and low cost	Low purity and low specificity	Ryu et al. (2020), Martinez-Greene et al. (2021)

### 2.4. Characterization techniques

The characterization of sEVs is essential for understanding the characteristics, structure, and function of sEVs as they affect drug loading and drug delivery (Kimiz-Gebologlu and Oncel, 2022). Numerous methods are available to verify the size, concentration, and purity of sEVs. The Minimal Information for Study of Extracellular Vesicles (MISEV) 2018 guidelines recommend the following steps for characterization: evaluation of sEV markers using two techniques (e.g., electron microscopy/imaging and nanoparticle tracking analysis/dynamic light scattering) and the identification of contaminants in sEV preparations by western blotting or flow cytometry, and the quantification of sEV preparation (e.g., protein concentration to particle concentration ratio) (Thery et al., 2018).

The MISEV2018 guidelines recommended that the characterization of sEVs by protein require at least three positive protein markers, including at least one transmembrane/lipid binding protein and at least one negative protein marker (Thery et al., 2018). Although protein blotting is a simple and widespread method for the analysis of sEVs, it has limited specificity, lacks multiplicity, offers only a small amount of information for a large number of sEV proteins, and does not exclude contaminants from other vesicles (Doyle and Wang, 2019; Zhang et al., 2019). To analyze the concentration and size of sEVs, one can choose between nanoparticle tracking analysis (NTA) and tunable resistive pulse sensing (van der Pol et al., 2014; Kurtjak et al., 2022). However, these methods cannot distinguish among EVs, protein aggregates, and lipoproteins, so not all small particles are quantified. Recently, a microstructured fiber-assisted nanoparticle tracking analysis (FaNTA) has been developed, which allows for the separation of all types of nano-objects less than 20 nm in diameter by recording the ultra-long trajectories of rapidly diffusing polydisperse nanoparticles, greatly improving the criteria for accurate characterization of monodisperse and polydisperse nanoparticle samples (Nissen et al., 2022). Dynamic light scattering (DLS) can be used to determine the size distribution of EVs, but cannot distinguish between EVs, protein lipoproteins, or other particulate matter (Haney et al., 2015). Electron microscopy enables visualization of sEVs to accurately assess sEV morphology and quality, as well as any other co-isolated contaminants such as proteins, DNA, lipoprotein, or viruses (Noble et al., 2020). Flow cytometry can also be used to analyze sEVs but it requires a separate suspension of particles to detect sEVs, which can lead to inaccurate data when the concentration of sEVs is too high or when sEVs are clustered together (Szatanek et al., 2017; Doyle and Wang, 2019). Nanoflow cytometry techniques have also been developed, which analyze individual sEVs with improved resolution and allow for multi-parameter measurements (Padda et al., 2019). Nanoflow cytometry shows great promise in the analysis of sEVs, but is still in the developmental stage and appropriate instrumentation, antibodies, and controls must be used to ensure accurate sEV detection (Morales-Kastresana et al., 2019).

Additionally, there are several ways to characterize sEVs. Smith et al. (2015) used Raman spectroscopy, a technique based on the illumination of a sample by a laser, to study the chemical composition of individual sEVs. Moreover, Zhao et al. (2016) developed a microfluidic epifluorescence search chip that is easy to handle and detects three sEV tumor markers (CA-125, EpCAM, and CD24). This design allows the detection of sEVs at a limit of 750 particles/μl, which is 1,000 times more sensitive than conventional methods such as protein blotting (Zhao et al., 2016).

### 2.5. Storage of sEVs

Although the standard indications for the storage of biological samples or sEVs are no longer provided in the MISEV2018 guidelines (Thery et al., 2018), improper storage methods can affect the concentration, physical properties, and function of sEVs (Wu et al., 2021; Gelibter et al., 2022). Therefore, it is both important and necessary to explore more appropriate storage conditions. For example, at present, sEVs are commonly stored in phosphate buffer (PBS) at −80°C without freeze-drying or spray-drying (Wu et al., 2021). Gelibter et al. (2022) conducted three sets of experiments: in the first set of experiments, they proved that under −80°C storage conditions, the concentration and purity of sEVs decreased continuously with increasing time, the particle size increased, and the Zeta potential changed; in the second set of experiments, they found that either a fast or slow freeze–thaw cycling mode resulted in a gradual increase in particle size in a cycle-dependent manner; and in the third set of experiments, they confirmed that the freeze–thaw process resulted in the rupture of the sEVs films, which were subsequently re-gelatinized into new particles (Gelibter et al., 2022). Gorgens et al. (2022) also confirmed that the −80°C storage method in PBS is suboptimal, and instead found that PBS supplemented with human albumin and trehalose (PBS-HAT) significantly improved the short- and long-term preservation of sEVs in samples stored at −80°C, maintaining stability over multiple freeze–thaw cycles.

Therefore, fresh sEVs should be used whenever possible, but if long-term storage is required, repeated freeze–thawing of samples must be avoided, and storage in PBS-HAT is preferred.

## 3. sEVs as DDS

To date, numerous researchers have explored sEV-based DDS for therapeutic purposes. For example, Sun et al. (2010) found that sEV-containing curcumin enhanced the anti-inflammatory activity of curcumin by intraperitoneal injection in a lipopolysaccharide (LPS)-induced septic shock mouse model, which may be attributed to the increased stability and concentration of curcumin in the blood by sEVs. Lai et al. (2020) demonstrated that peripherally injected sEVs can deliver miRNAs to the brains of mice with subarachnoid hemorrhage (SAH), the underlying mechanism of which is regulated by neuroinflammatory regulation. Thomas et al. (2021) assembled WNT3a on sEVs, which actively penetrated intact cartilage for efficient delivery into cartilage, contributing to the healing of osteochondral defects for therapeutic purposes.

The properties of sEVs make them ideal for drug delivery; however, how to effectively load drugs into sEVs is a challenge that remains to be addressed for targeted therapeutic applications. For this purpose, researchers have established many platforms, including incubation, electroporation, ultrasound treatment, extrusion, freeze–thaw cycling, and saponins (Table 3). For example, Sun et al. (2010) found that sEV-containing curcumin, which was obtained by incubating sEVs with curcumin, ameliorated LPS-induced septic shock and suggested that this method can be used to elevate the concentration of curcumin in sEVs. Yan et al. (2022) successfully encapsulated miR-31-5p mimics into milk-derived sEVs by electroporation and demonstrated that miR-31-5p loaded into sEVs achieved higher cellular uptake and was degradation-resistant. Hajipour et al. (2021) used ultrasound treatment to effectively (40.55 ± 4.21%) load human chorionic gonadotropin into sEVs isolated from uterine fluid. It is important to note that the results of drug delivery studies are difficult to generalize because the results are highly dependent on sEVs sources, isolation techniques, therapeutic agents, and drug delivery protocols (Walker et al., 2019). A study has been found that electroporation and transfection of sEVs to load the short interfering RNA (siRNA) results in the disruption of sEVs, precipitation of siRNA on membranes, and a loss of cargo delivery capability (McCann et al., 2022). Recently, a new technique was described to integrate siRNA sequences into a Dicer-independent pre-miRNA stem loop (pre-miR-451), using a miRNA sorting mechanism that improves the loading efficiency of sEVs. The therapeutic dose required by this siRNA delivery method is at least ten times less than that required for siRNA delivered via lipid nanoparticles, greatly reducing the possible toxicity (Reshke et al., 2020). This suggests that effective drug packaging has a multiplier effect on therapy. Furthermore, putting the desired small-molecule or nucleic-acid drugs into sEVs in a way that does not disrupt the cell membrane or the biological activity of the cargo is the main challenge at hand. Meanwhile, common sEVs loading strategies often result in low loading efficiency (<30%) compared to synthetic nanomedicines (Guo et al., 2021). Kooijmans et al. (2013) suggested that when electroporated, siRNA forms extensive aggregates and has a substantial retention rate of less than 0.05% in sEVs. To avoid this, Guo et al. (2021) developed a facile magnetic extrusion method for the preparation of endosome-derived vesicles. The high yield and consistency of this method somewhat helped to overcome the difficulties faced when sEVs were loaded.

**TABLE 3:** Comparison of the advantages and disadvantages of sEV loading methods.

Methods	Advantages	Disadvantages	References
Incubation	Simple method, low cost, and minimal damage	Low sample loading efficiency	Sun et al. (2010), Xu and Xu (2021), Seo et al. (2022)
Electroporation	High sample loading efficiency	Damage membrane integrity	Burgio et al. (2020), Mehryab et al. (2020)
Ultrasound treatment	High sample loading efficiency and long drug release duration	Not suitable for large-scale applications	Kim et al. (2016), Luan et al. (2017)
Extrusion	High sample loading efficiency	Cytotoxicity	Fuhrmann et al. (2015), Xi et al. (2021)
Freeze-thaw cycle	Moderate loading efficiency	sEV aggregation	Hajipour et al. (2021), Xi et al. (2021)
Saponins	High sample loading efficiency	Cytotoxicity	Haney et al. (2015), Xi et al. (2021)

How to prolong the residence time in circulation and increase sEVs targeting ability are remaining challenges to be addressed (Xu and Xu, 2021). The intact transmembrane proteins (CD81 and CD9) and integrins (CD51 and CD61) in sEVs are thought to have some homing and targeting functions, but their targeting ability remains too weak for application (Fitzner et al., 2011). Furthermore, some components of naturally isolated sEVs are not essential for drug delivery, and synthetic therapeutic biomaterials (e.g., modified and synthetic sEVs) are necessary for a more pure and efficient characterization of nanocarriers (Garcia-Manrique et al., 2018; Claridge et al., 2021). Hence, modification of sEVs is essential for their biodistribution, half-life, ability to target specific cells, and therapeutic potential (Man et al., 2020). Various engineering approaches can be used to modify homing peptides or ligands on the surface of sEVs, which can confer targeting capabilities to sEVs and thus increase their therapeutic efficiency (Luarte et al., 2016; Xu et al., 2021). Modification strategies include genetic engineering and chemical modifications (Alvarez-Erviti et al., 2011). For example, researchers have found that the RVG peptide (TIWMPENPRPGTPCDIFTNSRGKRASNG) selectively binds to acetylcholine receptors and can be used to develop neuro-specific sEVs for drug delivery to the CNS (El-Andaloussi et al., 2012). Moreover, Yu et al. (2021) used genetic engineering techniques to target donor cells to the RVG peptide of α7-nAChR and added highly specific variants capable of degrading amyloid beta (Aβ) to the surface of sEVs. Didiot et al. (2016) demonstrated that sEVs loaded with hydrophobically modified small interfering RNA (hsiRNA) targeting Huntington protein mRNA were effectively internalized by mouse primary cortical neurons and promoted dose-dependent silencing of Huntington mRNA and protein. Cui et al. (2019) constructed RVG-coupled sEVs derived from MSCs by chemical coupling. They found that this approach enhanced the binding capacity of sEVs to the cortical and hippocampal region of the AD mouse model and prevented memory deficits by inactivating astrocytes and balancing the inflammatory response.

In addition, sEVs may have some risks as drug carriers for ND. Indeed, some studies have shown that sEVs accelerate the fibrosis of proteins such as a-synuclein and Aβ, depending on the composition of the lipids (Grey et al., 2015; Stuendl et al., 2016; Lindberg et al., 2017). Furthermore, Grey et al. (2015) used mass spectrometry to identify several phospholipids in sEVs, including phosphatidylserine, phosphatidylcholine, phosphatidylinositol, phosphatidylethanolamine, and the gangliosides GM2 and GM3. Vesicles containing ganglioside GM1 or GM3 were found to accelerate α-synuclein aggregation, and α-synuclein is highly sensitive to pH. At the resting state, α-synuclein aggregation is very slow at neutral pH, while at weakly acidic pH (<6), α-synuclein aggregation is significantly accelerated. Therefore, we believe that caution should be taken to maintain a neutral pH when using sEVs as drug carriers for treating ND.

Although these studies have explored the use of sEVs as DDS, sEVs still cannot meet the demand as DDS. Indeed, sEV-based drug delivery lacks appropriate guidelines pertaining to isolation and purification; in addition, there are deficiencies in the perception of sEVs as a DDS mechanism, inadequate clinical-grade production, and the fact that cell types that could be ideal sources of drug-delivery-grade sEVs have not been identified (Meng et al., 2020). Furthermore, a protein corona is formed by the adsorption of serum proteins onto vesicles during circulation, limiting vesicle migration and affecting DDS targeting. It has been shown that the formation of a protein corona alters the physicochemical properties of vesicles, giving them biological properties that differ from their synthetic properties (Walkey and Chan, 2012; Hadjidemetriou et al., 2015; Skliar et al., 2018; Toth et al., 2021). Therefore, in the future, nanomedicines must be designed that are either completely resistant to plasma components or promote the binding of specific plasma components, thereby improving circulatory or specific targeting properties, respectively.

## 4. Route of administration

The route of administration also has an important impact on the biodistribution, therapeutic efficacy, and short/long-term biological effects of sEVs. Currently, the main methods of administration for treating ND include intravenous injection, nasal administration, oral administration, and stereotactic injection (Xu et al., 2021). The first three modalities have high patient compliance, whereas stereotactic injections are often not accepted by the general public because of their invasive nature. In this regard, several researchers have also conducted relevant studies. For example, Cui et al. (2019) found that sEVs could be tracked in the brain by intravenous injection and significantly improve learning and memory capacity, reduce plaque deposition and Aβ levels, and normalize inflammatory cytokine levels. Peng et al. (2022) designed a nanocarrier with a targeting function that combines therapeutic MSC-derived sEVs with curcumin to target drug transport into the cytoplasm of target cells after intranasal administration, thereby significantly improving the motor and coordination abilities of PD model mice. Although oral administration is also well accepted by the public, it is rarely used in research because of the first-pass effect, the enzymatic disruption of the structural integrity of sEVs, and the difficulty of diffusing sEVs to the brain (Agrawal et al., 2017). Here, we propose a scenario: if peptides capable of targeting the brain and sEVs are coupled to make emulsifiers, could the drawbacks be solved and used in clinical applications? Aqil et al. (2017) developed sEVs containing curcumin by loading curcumin into milk-derived sEVs. sEVs curcumin increased the drug content by 3–5 times compared with free curcumin when administered orally. Recently, there have been some reports on sEVs of plant origin. For example, some researchers have reported that broccoli-derived EVs have therapeutic potential due to their ability to transport exogenous miRNA (Del Pozo-Acebo et al., 2022). However, it has only been possible to demonstrate that miRNA can be taken up by human colorectal adenocarcinoma cells (Caco-2) *in vitro*, and whether EV uptake is altered upon digestion when cells are exposed to digested EVs is not yet known and requires further study (Del Pozo-Acebo et al., 2022). Nonetheless, it cannot be denied that this study holds great promise for pharmacological applications. Interestingly, Zhao P. et al. (2020) found that the amount of indocyanine green (ICG) PLGA nanoparticles in the brain following subcutaneous injection was more than 44-fold higher than the intravenous route. Additionally, Nazimek et al. (2020) compared the therapeutic effects of sEVs administrated via intravenous (iv), intraperitoneal (ip), intradermal (id), and oral administration on delayed-type hypersensitivity (DTH) induced by ovalbumin, and found that the strongest effect was observed with orally administered sEVs. This result partially reflects the importance of the route of administration.

Although these routes of administration have been studied, some shortcomings still need to be addressed. For example, the half-life index of sEVs with intravenous administration is too short and they are readily cleared by macrophages (Liu et al., 2017). Nasal administration also has some disadvantages including relatively small doses, limited olfactory epithelial surface area, the short retention time for drug absorption, and its effect on nasal secretions (Erdo et al., 2018). The choice of a more appropriate route of drug delivery still needs to be explored.

## 5. The theoretical basis of sEVs for ND treatment

Numerous articles have shown that neuronal cells in the CNS regulate the function of each other by releasing large amounts of sEVs (Fruhbeis et al., 2012; Sharma et al., 2019). These suggests that sEVs may play an important role to maintain the communication and homeostasis in the CNS. For example, sEVs are involved in controlling neurogenesis (Sharma et al., 2019), regulating myelin biogenesis (Bakhti et al., 2011), and repairing damaged neurons (Pei et al., 2019). Fruhbeis et al. (2013) found that activity-dependent release of the neurotransmitter glutamate triggered the secretion of oligodendrocyte-derived sEVs and that neurons could internalize these sEVs via endocytosis to improve neuronal viability under cellular stress. This suggests that oligodendrocyte-derived sEVs are involved in a novel mode of bidirectional neuron-glia communication that contributes to neuronal integrity. Wang et al. (2011) suggested that synaptophysin, a glycoprotein associated with astrocyte sEVs, may play a role in mediating neuroprotection and stimulating axonal growth. In addition, a number of articles have shown that sEVs can have a neuroprotective effect. For example, Haney et al. (2015) administrated sEVs-containing peroxidase to the brain of the PD mouse model and found that these sEVs could protect SNpc neurons from acute oxidative stress. Kalani et al. (2016) found that embryonic stem cell-derived sEVs loaded with curcumin could also induce neuroprotective effects in an ischemia-reperfusion injury model. Similarly, sEVs secreted by adipose-derived stem cells are neuroprotective. In AD, sEVs secreted by adipose-derived MSCs have potent neuroprotective effects against Aβ-induced neuronal toxicity, reducing neuronal damage, and promoting neurogenesis (Ma et al., 2020). Adipose-derived stem cells secreting sEVs improved motor performance and reduced glial cell activation in an animal model of amyotrophic lateral sclerosis (ALS) (Bonafede et al., 2020). Although sEVs are involved in neuroprotective processes, they are also involved in the pathogenesis of CNS disease (Venturini et al., 2019). For example, sEVs delivered some pathogenic proteins such as Aβ, α-synuclein, and mutant Huntington’s protein (mHTT) into healthy neuronal cells and induced dysfunction of receipt cells (Eitan et al., 2016; Stuendl et al., 2016; Xia et al., 2022). Therefore, it is important to fully understand the role of sEVs in the physiology and pathology of CNS. In parallel, these studies also indicated that sEVs can be used to treatment of NDD by regulating the biosynthesis, released or modified the contents in sEVs with gene editing.

## 6. sEVs in ND clinical applications

**FIGURE 3:**
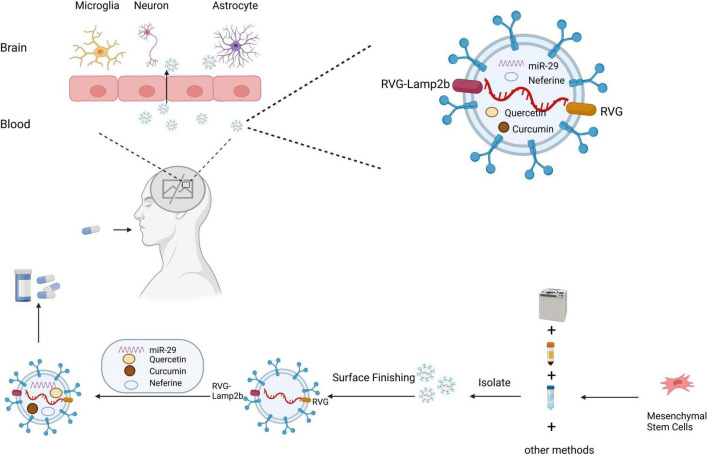
Schematic diagram of the clinical application of small extracellular vesicles as a drug delivery system in neurodegenerative diseases. Small extracellular vesicles have greater advantages as drug delivery systems due to their biological properties, which protect their contents from phagocytosis, and their ability to cross the blood–brain barrier. Small extracellular vesicles are isolated by ultracentrifugation, density gradient centrifugation, and other methods. Small extracellular vesicles can be targeted to cross the blood–brain barrier for treating neurodegenerative diseases through surface modification and drug loading.

Due to the presence of the BBB and the complexity of the CNS, treatment of ND often requires consideration of whether the drug can effectively reach the brain tissue (Figure 3). As sEVs have the advantages of low immunogenicity and stable and long distance transport, they are considered the most promising tool for ND treatment at present (Loch-Neckel et al., 2022). sEVs also have great potential as non-invasive diagnostic biomarkers for ND and have been extensively reported in the literature. However, the current review focuses on the therapeutic application of sEVs rather than their diagnostic potential. The following sections focus on treatment methods that use sEVs loaded with therapeutic ingredients for ND.

### 6.1. Alzheimer’s disease

Alzheimer’s disease is a neurodegenerative disorder that usually develops in old age and is the most common form of dementia worldwide. The number of people with AD is increasing rapidly owing to the aging of the population (2022). The main clinical manifestations of AD are memory loss, cognitive decline, behavioral disturbances, and death from complications 10–20 years after the onset of the disease (Bartus et al., 1982). Three major pathological features are present in the brains of patients with AD patients, including Aβ-containing protein, neurofibrillary tangles containing tau protein, and plaque accumulation with progressive synaptic and neuronal loss (Castello et al., 2012).

BACE1 is a protease responsible for the N-terminal cleavage of amyloid precursor protein (APP), which produces aggregates to form Aβ polypeptides (Taylor et al., 2022). In view of this, researchers delivered sEVs-containing siRNA into the brains of AD mice via tail vein injection and found a significant decrease in the level of mRNA and protein of the therapeutic target BACE1, representing the first study to explore sEV-based drug delivery for CNS diseases and demonstrating the validity of using siRNA to deliver sEVs to treat ND (Alvarez-Erviti et al., 2011). Subsequently, scientists conducted numerous studies on the effects of sEVs in AD. For example, Morel et al. (2013) found that miR-124a delivered via sEVs enhanced the expression of excitatory amino acid transporter protein 2 (GLT1) on the surface of astrocytes, thereby regulating synaptic activity and improving glutamate uptake. This approach is expected to alleviate neuronal apoptosis in AD. Improving Aβ clearance is an effective strategy for AD treatment. Yuyama et al. (2012) found that increased secretion of sEVs with sphingomyelin synthase 2 (SMS2) siRNA significantly reduced extracellular levels of Aβ by enhancing Aβ uptake into microglia. Falker et al. (2016) found that PrP(C) enriched on sEVs from SH-SY5Y reduced the neurotoxicity of Aβ via promoting Aβ fibrosis. Jahangard et al. (2020) injected miR-29-containing sEVs to the CA1 (cornu ammonis area) of the hippocampus in a rat model of AD and found that miR-29-containing sEVs could partially recover cognitive deficits. Based on these studies, we suggested that sEVs that are modified by genetic engineering may be of potential therapeutic value for treating AD. Likewise, there are also some studies of sEVs loaded with drugs. Indeed, Wang et al. (2019) designed sEVs as carriers of the anti-inflammatory agent curcumin and effectively delivered curcumin to the BBB to inhibit Tau phosphorylation and prevent neuronal death via the AKT/GSK-3β pathway, thereby alleviating AD symptoms. Qi et al. (2020) treated AD models with free quercetin and quercetin-loaded sEVs, and found that the sEV delivery system improved quercetin permeability in the brain parenchymal tissue and improved cognitive function more than free quercetin. Tang et al. (2022) reported that sEVs encapsulated with neferine reduced the levels of Aβ in the brain and eased motor deficits in APP/presenilin1 (PS1) double transgenic mice.

### 6.2. Parkinson’s disease

Parkinson’s disease is a neurological disorder caused by degenerating dopaminergic neurons in the striatum nigra and the formation of Lewy bodies, with the main clinical manifestations being motor symptoms such as myotonia, resting tremor, bradykinesia, postural balance disorders, and non-motor symptoms such as autonomic dysfunction, mood disorders, and sleep disorders (Samii et al., 2004; Zhang et al., 2005).

Small extracellular vesicles have also been used in the treatment of PD via different pathways. For example, Chen H. X. et al. (2020) found that sEVs from umbilical cord MSCs could inhibit apoptosis of DA neurons by inducing autophagy in a model of PD, with potential therapeutic effects. In addition to sEVs of natural origin, modified sEVs can be used for treating PD. For example, Cooper et al. (2014) and Izco et al. (2019) modified sEVs by combining brain-specific RVG with siRNA or shRNA minicircles to treat PD. They found that these sEVs reduced the levels of α-synuclein mRNA and protein throughout a mouse brain 7 days after peripheral injection, and downregulated α-synuclein aggregation in a mouse model of PD. Haney et al. (2015) and Kojima et al. (2018) found that peroxidase-loaded sEVs induced neuroprotective effects *in vitro* and *in vivo* models of PD. Additionally, sEVs-containing drugs can significantly enhance the effects of existing drugs. For example, Qu et al. (2018b) loaded dopamine into blood sEVs by incubation and delivered to the brain, demonstrating that the dopamine distribution in the brain was increased more than 15-fold, with good therapeutic efficacy. Liu et al. (2020) designed a curcumin analog-based nanoscavenger (NanoCA) that stimulates nuclear translocation of the major autophagy regulator transcription factor EB (TFEB), triggering autophagy and calcium-dependent secretion of sEVs to clear α-synuclein.

### 6.3. Huntington’s disease

Huntington’s disease is inherited in an autosomal dominant manner and is caused by the amplification of the cytosine-adenine-guanine (CAG) trinucleotide repeat sequence in the short arm of the coding region of the HD gene (located at 4p16.3) in exon 1 of the Huntington gene (MacDonald et al., 1993). A higher number of CAG repeats is also thought to be associated with earlier disease onset, faster clinical progression, and increased disease severity (Aziz et al., 2009). Clinical features of HD include progressive motor dysfunction (mainly chorea), cognitive decline, and psychiatric disorders, such as personality changes and depression (Walker, 2007). Dysphagia is one of the most common and important complications of HD, often leading to aspiration pneumonia and death (Schumann-Werner et al., 2021).

Many studies have investigated the use of sEVs as DDS for HD treatment. For instance, Lee et al. (2016) found that adipose-derived stem cell sEVs (ASC-sEVs) significantly reduced the aggregation of mHTT in neuronal cells, upregulated the expression of PGC-1 and phosphorylated CREB, and reduce mitochondrial dysfunction and apoptosis, suggesting that ASC-sEVs have potential for treating HD. Hong et al. (2017) injected astrocytic-derived sEVs into the striatum of HD 140Q knock-in (KI) mice, demonstrating a reduced density of mHTT aggregates. Interestingly, the presence of mHTT protein was not detected in sEVs released from primary astrocytes in 140Q KI mice, highlighting the potential use of astrocyte-derived sEVs in HD treatment (Hong et al., 2017). In addition to studies on sEVs of natural origin, numerous studies have investigated engineered sEVs. For example, Didiot et al. (2016) found that hydrophobic modification of sEVs mediated siRNA silencing of Huntington mRNA, which is expected to facilitate the development of therapeutic approaches for HD and other NDs. Zhang et al. (2021) designed a cytomegalovirus promoter-directed genetic circuit encoding the RVG tag and mHTT siRNA targeting sEVs and delivered the mHTT siRNA to the cortex and striatum via the sEVs circulatory system, showing that levels of mHTT protein and toxic aggregates were successfully reduced in the cortex and striatum of a mouse model of HD, providing a novel idea for the treatment of HD.

### 6.4. Other type of NDs

Other types of NDs, such as epilepsy, ALS, multiple sclerosis (MS), and spinal cerebellar ataxia (SCA), especially SCA, have been poorly studied.

Mesenchymal stem cell-derived sEVs administered intranasally are capable of treating the symptoms of LPS-induced epilepsy in mice. Long et al. (2017) found that the administration of sEVs following status epilepticus greatly reduced microglial activation, decreased overall neuronal losses in the hippocampus, and prevented cognitive and memory declines in the chronic phase. ALS is most commonly studied using transgenic mice overexpressing a reported G93A mutation of the human SOD1 gene [SOD1 (G93A)] (Vinsant et al., 2013). Bonafede et al. (2020) found that repeated administration of ASC-sEVs significantly improved motor function in treated SOD1 (G93A) mice and protected motor neurons, neuromuscular junctions, and muscles as well as reduced glial cell activation, demonstrating the potential therapeutic role of ASC-sEVs in human ALS. Wu et al. (2022) isolated sEVs from mouse neural stem cells and modified them to enable targeted delivery to the lesioned areas of MS model mice. The encapsulation of Bryostatin-1 (Bryo) in sEVs exhibited potent therapeutic effects compared to Bryo alone, suggesting that targeted delivery of Bryo based on sEVs significantly improves myelin protection and promotes myelin regeneration. You et al. (2020) found that MSC-derived sEVs reduced the loss of Purkinje cells and cerebellar myelin sheaths, alleviated neuroinflammation, and improved motor functions in SCA3 mice, which is expected to facilitate the development of SCA3 and other types of SCA.

Stem cell therapy has been a hot research topic in disease treatment recently, with several reports on this therapy. Coenzyme Q10 (CoQ10) is an electron donor in the mitochondrial electron transport chain and acts as an antioxidant to enhance mitochondrial function and prevent oxidative stress (Manzar et al., 2020). It has been demonstrated that CoQ10 has neuroprotective effects in ND and inhibits the pathological progression of neuroinflammation-related diseases (Ghasemloo et al., 2021). Sheykhhasan et al. (2022) found that sEVs obtained from adipose-derived stem cells could act as carriers of CoQ10. CoQ10 delivered by sEVs enhanced cognitive and memory deficits in AD by increasing Brain-derived neurotrophic factor (BDNF) and sex-determining region Y-box 2 (SOX2) levels in the hippocampus, thereby increasing the therapeutic effect of CoQ10. This therapeutic effect is not only due to the delivery of CoQ10 but also to the potential of stem cell-driven sEVs in tissue regeneration. Additionally, Losurdo et al. (2020) demonstrated that the intranasal delivery route of sEVs from cytokine-preconditioned MSCs could induce immunomodulatory and neuroprotective effects in triple-transgenic 3xTg mice. As a result, sEVs derived from stem cells can perform a dual protective function when used as drug delivery vehicles, aiding healing via their own therapeutic properties, thus creating a microenvironment that promotes healing while also serving as a targeting vehicle for the delivery of appropriate drugs.

## 7. Limitations of sEVs as drug delivery systems

The use of sEVs as a therapeutic approach for ND has largely focused on their functional aspects, but their negative effects have rarely been studied. Although sEVs can pass through the BBB, the amount that can pass is limited (about 5%) and the remaining 95% may remain at unknown sites of action and cause systemic adverse effects (Sonvico et al., 2018). Safety is also a key consideration, as nanomaterials may cause oxidative stress, apoptosis, and inflammation in the brain (Vega-Villa et al., 2008). Therefore, future designs should consider the biodegradability of sEVs from the brain. Additionally, the protein corona formed on the surface of sEVs may shield its surface from targeting molecules and lead to targeting errors (Salvati et al., 2013). The development of sEVs with long blood circulation times is also extremely challenging. Most of the targeted sEVs take a long time to penetrate the BBB and reach the target site, so they tend to accumulate in the liver and spleen (Chopra et al., 2022).

Many studies on sEVs have been limited to *in vitro* or animal model-level studies, and no further *in vivo* studies have been conducted to assess the safety of sEV therapeutic approaches. In previous studies, sEVs have been shown to play a role in promoting neurogenesis, inhibiting neuroinflammation, promoting angiogenesis, and promoting synaptic plasticity. However, not all effects are relevant for treating a disease, and some even pose significant risks, such as whether the treatment of non-oncological diseases increases the risk of cancer promotion. Despite the great therapeutic potential of sEV-based technologies, they are still in the growth phase. Most current research on sEVs is basic research and rarely used in the clinic, due to the fact that bench-to-bedside translational techniques typically require reasonable scalability, throughput, and thorough validation to screen large numbers of clinical samples (Li P. et al., 2017). For example, the amount of sEVs needed to be injected into a patient to achieve a therapeutic dose depends on several factors, such as the source, type and amount of sEVs, the purpose of the treatment, and the patient’s condition. Usually, sEVs injections require strict dose calculation and control, with individualized adjustments based on individual differences in patient weight, age, and condition. In addition, sEVs treatment also requires rigorous safety assessment and monitoring to ensure treatment efficacy and safety (Chen et al., 2023). To ensure the safe and effective clinical use of sEVs for treating major diseases, their safety and long-term effects should be further explored. Therefore, a comprehensive testing program for sEVs, including pharmacokinetic, biodistribution, and acute and chronic toxicity testing, is necessary prior to their introduction into clinical trials.

## 8. Conclusion and future outlook

Small extracellular vesicles are widely used in brain-targeted delivery systems due to their low toxicity, low immunogenicity, high stability, high delivery efficiency, high biocompatibility, and trans-BBB function. sEVs can participate in inter-neuronal information exchange through self-delivery, endocytosis, or donor-receptor-specific binding for brain-targeted drug delivery. sEVs can also be chemically or genetically modified for efficient brain targeting. Today, sEVs are widely used in the diagnosis and treatment of ND and have become the most promising DDS.

However, the clinical application of sEVs in ND is still challenged by many factors, such as the lack of standardization and clinical utility of purification and detection methods, lack of sensitivity and specificity of screening indicators, the incomplete elucidation of the mechanism of action of sEVs targeting into the brain for expression and regulation, and the safety and efficiency of drug delivery. Although engineered sEVs have shown great potential in ND, clinical mass production remains a pressing issue. Furthermore, to better understand the *in vivo* behavior of sEVs, assessment of their pharmacokinetic properties is crucial. Analysis of the *in vivo* distribution of sEVs is a prerequisite for developing sEVs-based therapeutics and drug delivery vehicles that can accurately predict therapeutic doses and potential side effects. Therefore, the comprehensive screening of sEVs using sensitive and reliable measurement methods, the precise analysis of the relevant signaling pathways and molecular mechanisms, the pharmacokinetic properties of sEVs, and the selection of the correct and effective markers and therapeutic targets remain the focus of future research.

Today, many biotech companies are contributing to the field of sEVs, such as Evox therapeutics, which is developing modified sEVs to improve their ability to deliver siRNA across the BBB (Cully, 2021). Additionally, Evox is optimizing the sEVs delivery of proteins or mRNA to rare diseases within the CNS and other organs (Cully, 2021). Researchers have been exploring and continuously optimizing sEVs as potential therapies for various diseases. For example, Liang et al. (2022) employed an engineering strategy to decorate albumin onto the surface of the sEVs through surface display of albumin binding domains to prolong the circulation time of sEVs in the body and to reduce the frequency of sEVs administration. In conclusion, sEVs still offer hope for patients with ND and have great clinical research value, providing a new approach to the diagnosis and treatment of ND and better elucidating the pathophysiological mechanisms.

## Author contributions

ZJ and RH designed the structure of the manuscript and finalized the manuscript. RP and DC drafted the manuscript. RP, DC, and LH provided critical revisions and improvements to the manuscript. All authors approved the final version of the manuscript.
